# Correlating the succession of microbial communities from Nigerian soils to petroleum biodegradation

**DOI:** 10.1007/s11274-023-03656-7

**Published:** 2023-07-01

**Authors:** Paul Iturbe-Espinoza, Matthijs Bonte, James T Weedon, Martin Braster, Bernd W Brandt, Rob JM van Spanning

**Affiliations:** 1 Systems biology lab, Department of Molecular Cell Biology, Faculty of Science, Vrije Universiteit Amsterdam, De Boelelaan 1085 (location code O|2-2E51), NL-1081HV Amsterdam, The Netherlands; 2 Department of Environmental Science, Aarhus University, Roskilde, Denmark; 3 Shell Global Solutions International BV, The Hague, The Netherlands; 4 MB-Water, Amsterdam, The Netherlands; 5 Department of Ecological Science, Faculty of Science, Vrije Universiteit Amsterdam, Amsterdam, The Netherlands; 6 Department of Preventive Dentistry, Academic Centre for Dentistry Amsterdam (ACTA), University of Amsterdam and Vrije Universiteit Amsterdam, Amsterdam, The Netherlands

**Keywords:** Degradation capacity, Oil biodegradation, PAHs, Pre-exposure, Soil microbial communities

## Abstract

Whilst biodegradation of different hydrocarbon components has been widely demonstrated to occur by specialist oil-degrading bacteria, less is known about the impact on microbial communities as a function of oil composition by comparing the biodegradation of chemically complex fuels to synthetic products. The objectives of this study were (i) to assess the biodegradation capacity and succession of microbial communities isolated from Nigerian soils in media with crude oil or synthetic oil as sole sources of carbon and energy, and (ii) to assess the temporal variability of the microbial community size. Community profiling was done using 16 S rRNA gene amplicon sequencing (Illumina), and oil profiling using gas chromatography. The biodegradation of natural and synthetic oil differed probably due to the content of sulfur that may interfere with the biodegradation of hydrocarbons. Both alkanes and PAHs in the natural oil were biodegraded faster than in the synthetic oil. Variable community responses were observed during the degradation of alkanes and more simple aromatic compounds, but at later phases of growth they became more homogeneous. The degradation capacity and the size of the community from the more-contaminated soil were higher than those from the less-contaminated soil. Six abundant organisms isolated from the cultures were found to biodegrade oil molecules in pure cultures. Ultimately, this knowledge may contribute to a better understanding of how to improve the biodegradation of crude oil by optimizing culturing conditions through inoculation or bioaugmentation of specific bacteria during ex-situ biodegradation such as biodigesters or landfarming.

**Supplementary Information:**

The online version contains supplementary material available at 10.1007/s11274-023-03656-7.

## Introduction

Hydrocarbons are ubiquitous in the environment as a result of both natural seeps and accidental spills of crude oil and related substances. Though biodegradation of different hydrocarbon components by specialized oil-degrading bacteria has been widely demonstrated (Ławniczak et al. 2020), little is known about the responses of microbial communities to changes in the oil composition. There is a gap in understanding on how biodegradation of chemically complex fuels compares to synthetic products which are made up of a much smaller amount of hydrocarbon constituents (and lack trace elements like sulfur or heavy metals). The global interest in synthetic hydrocarbon fuels has been steadily increasing as a means to mitigate CO_2_ emissions associated with the processing and utilization of fossil fuels (Li et al. 2016; Peter 2018). For example synthetic kerosene for aviation (made from H_2_ and CO_2_) (Zoller et al. 2022) or solar fuel from the photocatalytic reduction of CO_2_ (Gong et al. 2022). Biodegradation of synthetic oil is mentioned in literature (Mouradian et al. 2023), but it has not been compared to biodegradation of natural oil yet. To our knowledge, this is the first study comparing the biodegradation of natural versus synthetic oil.

Crude oil is a complex mixture of hydrocarbons, comprising a wide variety of linear, branched, cyclic and aromatic structures, and of non-hydrocarbon fractions, such as resins and asphaltenes ((Brown et al. 2017a). A key concern is the toxicity and carcinogenesis of polycyclic aromatic hydrocarbons (PAHs) (Goldman et al. 2001; Lee and Dong 2011), which have more than two fused rings in their structure. PAHs are present in many types of crude oil at varying concentrations: for example, PAH content is very low in Bonny crude oil (0.01 wt %) ((Brown et al. 2017b), more typical concentrations are around 0.83 wt % such as seen in crude oil from the North Sea (Aas et al. 2000), and concentrations as high as 1.47 wt % were observed in crude oil that leaked out of the Exxon Valdez (Deepthike et al. 2009).

At present, oil spills occur frequently in oil-producing regions, such as the Niger Delta in Nigeria. Pipeline sabotage, crude oil theft, operational spills, and artisanal refining in this region have all at various times contributed to devastating ecological disasters (Pegg and Zabbey 2013). Excavation and *ex-situ* treatment of oil-polluted soils is expensive and laborious. Physical and chemical methods are commonly used during the clean-up projects although they cannot remove hydrocarbons completely (Ghosal et al. 2016). Bioremediation of the oil by aerobic indigenous microorganisms from the oil-contaminated environments is a more promising method for the degradation of hydrocarbons (Azubuike et al. 2016; Ghosal et al. 2016; Iturbe-Espinoza et al. 2019).

Biodegradation of PAHs is challenging for bacteria because of the relatively high amount of energy required to break the covalent bonds of the aromatic rings. PAHs are thermodynamically more stable and resistant to microbial degradation due to the even distribution of electrons around the aromatic rings. Correlations among the environmental persistence, resistance to biodegradation, and numbers of benzene rings in the PAH structures are well documented (Banerjee et al. 1995). Low-molecular weight PAH (LMW PAH) compounds, containing two or three aromatic rings, are relatively easier to breakdown than the high-molecular weight PAHs (HMW PAHs), containing four or more aromatic rings. From this group, the degradation of HMW PAHs with five or more fused rings by bacterial genera is poorly understood and may require the cooperation of a more extended microbial consortium (Kanaly and Harayama 2010).

Soil bacterial communities may respond to oil contamination by simultaneous changes in total bacterial biomass, community composition, and functional capabilities (Iturbe-Espinoza et al. 2019). Past studies of soil microbial communities based on 16 S rRNA gene amplicon sequencing have provided valuable information on the taxonomic identity and relative abundances of bacterial operational taxonomic units (OTUs) during the degradation of crude oil (Allamin et al. 2020; Iturbe-Espinoza et al. 2022; Vita et al. 2022). However, the standard technique produces data for relative abundances of the taxa within the microbial community, making it difficult to discern patterns related to population sizes of individual OTUs and thereby makes it difficult to compare the community compositions and population sizes across samples of the same experiment. A few recent studies tackled this issue by adding an internal standard gene prior to DNA extraction to simultaneously measure soil bacterial abundances and community composition in soils (Smets et al. 2016). Although this method does not consider variation in the copy number of 16 S rRNA gene across strains and taxa, it provides valuable quantitative information about changes in absolute abundance of community members. In the present study, we applied this new approach to monitor bacterial communities more quantitatively. Correlating the absolute abundance of individual OTUs within microbial communities to the biodegradation of different types of molecules in crude oil will improve the identification of specialized alkane and PAH-degrading bacteria.

The objectives of this study were therefore (i) to assess the biodegradation capacity and succession of microbial communities isolated from Nigerian soils in media with crude oil or synthetic oil as sole sources of carbon and energy considering changes in total bacterial biomass, and (ii) to determine the preference of degradation of the different types of oil molecules by specialized oil-degrading consortia. For the latter, we designed a synthetic oil composed of a defined mix of alkanes plus the sixteen priority PAHs according to the US-Environment Protection Agency (EPA-PAHs). Because the degree of pre-exposure has a strong influence on biodegradation capacity (Okere et al. 2017; Nicholls et al. 2022), we used two different types of soil collected from the Niger Delta region of Nigeria as bacterial inoculum, each of them with a different degree of oil contamination. To measure the succession and temporal variability in the bacterial community size, we performed 16 S rRNA gene amplicon sequencing with genomic DNA of *Geobacillus kaustophilus* as internal control in order to convert relative abundances of the OTUs to quantitative abundances across samples. The results may contribute to a better understanding of consortia-mediated biodegradation of HMW PAHs as well as to identify organisms involved in the degradation of the different types of oil molecules. This information can support the design of remediation systems that rely on hydrocarbon biodegradation, for example through inoculation or bioaugmentation of specific bacteria (Gao et al. 2022) or by stimulating biodegradation in landfarming through tiling (Brown, et al. 2017) or addition of electron acceptors such as sulfate (Wei et al. 2018).

## Methods

### Crude oil and soil samples

The Shell Petroleum Development Company (SPDC) of Nigeria Ltd provided fresh Bonny Light crude oil and soil samples. Soil samples were taken from the surface (0–5 cm depth) in 5 mL Eppendorf tubes from a zone with different degrees of oil pollution before a cleanup program to remediate mangrove areas in the Niger Delta region of Nigeria (Bonte et al. 2020). We used two contaminated soil samples from this heavily impacted site where two oil spills happened in 2008 (Pegg and Zabbey 2013): a less contaminated soil (L-soil) that does not require active remediation and a more contaminated soil sample (M-soil) that requires remediation or a higher-tier risk assessment according to the Nigerian environmental legislation (EGASPIN 2018). The L-soil sample had a residual total petroleum hydrocarbon (TPH) of 4900 mg/kg and was collected at 4°38’30.6"N 7°15’23.4"E, in an area with 5% coverage of live mangroves, 34% moisture, < 1 mg/kg NO_3_^−^, 55% of oil surface. The M-soil sample had a residual TPH of 108,000 mg/kg and was collected at 4°37’39.3"N 7°15’47.1"E, in an area with dead mangroves and 15 cm of soft mud on top, 70% moisture, 12 mg/kg NO_3_^−^, 2% of oil surface (Bonte et al. 2020). The soil samples derived from a saline mangrove environment. Previous sampling showed salinity in surface water ranged between 6 and 23 ppt (Gundlach et al. 2021; Jane et al. 2022). Soil sampling locations were located topographically below the high tide level and the sites are inundated daily. The crude oil and soil samples were transported in a box cooled with cooling elements at around 4 °C for about two days from Nigeria to the Netherlands. The crude oil and soil containers were stored at 4 °C for further incubation experiments.

### Chemicals

For the incubation experiments, we used a stock solution of saturated alkanes from C_7_ to C_30_ (1,000 µg/mL each component; Supelco; catalog number 49,451-U) in hexane; and a stock solution of Polynuclear Aromatic Hydrocarbons Mix (2000 µg/mL for each component; Supelco; catalog number CRM47543) in benzene: dichloromethane (50:50). Both mixtures of hydrocarbons were purchased from Sigma-Aldrich.

### Incubation conditions

Soil microbial consortia were incubated in 30 mL serum bottles containing 10 mL of mineral media (200 mg MgSO_4_, 20 mg CaCl_2_, 1000 mg KH_2_PO_4_, 1000 mg K_2_HPO_4_, 1000 mg NH_4_NO_3_ per liter of distilled water, pH 6.9) previously sterilized in autoclave at 121ºC, 15 lb pressure for 15 min. Two different carbon substrates were used as carbon and energy sources: crude oil (fresh Bonny Light) and synthetic oil. The synthetic oil was composed of a defined mixture of alkanes (C_7_-C_30_) (5 µg/mL of each) and the EPA-PAHs (1 µg/mL of each). Because the concentration of PAHs in the crude oil was relatively low compared to other types of crude oil, we spiked the EPA-PAHs in the crude oil to a level that allowed us to follow biodegradation throughout the experiment with a sufficient degree of accuracy. 5 µL of the spiked crude oil was added to the serum bottles containing the mineral media resulting in a concentration of 0.5 µg/mL of each PAH. The serum bottles were incubated at 30 °C and shaken at 120 rpm overnight to allow the evaporation of the solvents (hexane, benzene and dichloromethane) before adding the soil. Around one milligram of soil was inoculated in the serum bottles containing mineral media and the two types of oil. We used one milligram of soil to increase the bioavailability of the oil molecules in the media, to promote the selection of hydrocarbon-biodegrading consortia, and to facilitate the hexane extraction of hydrocarbons. The bottles were closed with Teflon septa and crimp caps, incubated at 30 °C and shaken at 120 rpm to promote aerobic biodegradation of hydrocarbons considering that the degradation of 1 mg of oil pollutants needs roughly 3 mg of oxygen (Troquet et al. 2003). Thus, 5.3 mg of oxygen in the headspace of the serum bottles was likely to be sufficient for the aerobic biodegradation of approximately of 0.8 mg of synthetic oil. Media with the two different types of oil but without soil were used as weathering controls (W control). For the chemical and microbiological analyses, we used destructive sampling in triplicate at the start of the incubation, and additionally after one, two, three, and six months of incubation.

To test if single isolated species (as described below in 2.6) were able to degrade hydrocarbons, they were used as inocula in serum bottles containing mineral media and synthetic oil. These bottles were closed with PTFE serum stoppers (DWK Life Sciences Kimble™) to assure a closed system during incubation. The headspace CO_2_ concentration was measured (see below Sect. 2.5) throughout the experiment as an indicator of hydrocarbon biodegradation.

### Hydrocarbon analysis

Hydrocarbon residues were extracted using n-hexane as described earlier (Brown et al. 2018). NaCl (250 g/L) was dissolved in the media prior to extractions with around 1.2 mL of n-hexane (around 10% of the total volume of the media). The extractions were performed in 15 mL glass tubes. An aliquot of n-hexane was used to rinse the emptied serum bottles to recover leftover hydrocarbons. The n-hexane was then transferred into the test tube. The tubes were closed using PTFE/silicone cups. Mixing was achieved through vigorous shaking by hand by inverting the tubes around 50 times to dissolve the NaCl. The aqueous lower layer and organic upper layer were allowed to separate for 10 min before collection of the organic layer into a glass vial with PTFE/silicone septum. A second aliquot of n-hexane was used to rinse the bottle and repeat the extraction. The n-hexane solution containing the extracted hydrocarbons was adjusted to a final volume of 2 mL. 5α-androstane (2.5 µg/mL) (Sigma-Aldrich) was added as an internal standard.

We use a gas chromatography-flame ionization detector (GC-FID) to measure the concentration of hydrocarbons at each sampling time. The GC-FID analyses were performed on a Shimadzu model GC-2010 equipped with a column Zebron ZB-5 (30 m long x 0.32 mm I.D., 0.25 μm film thickness, 95% dimethyl- 5% diphenyl polysiloxane). The injection size of each sample was 2 µL. The injection was done in splitless mode with helium as carrier gas, linear velocity set to 40.0 cm/sec, and injector site temperature set to 290 °C. Separation of aliphatic and aromatic compounds was monitored with aliphatic and aromatic standards (Bennett and Larter 2000; Rodriguez et al. 2015). The temperature of the column was calibrated to simultaneously measure the n-hexane extractable alkanes and EPA-PAHs without previous fractionation to avoid loss of hydrocarbons (Supplementary Fig. 1). Its initial temperature was 60 °C with a hold time of 1 min. First, the temperature increased at 159.6 °C with a rate of 30 °C/min. Then, it increased to 184 °C with a rate of 4 °C/min with a hold time of 1 min. After that, it increased to 244.5 °C with a rate of 16 °C/min. It was then remained at 244.5 °C with a rate of 2 °C/min and with a hold time of 9.5 min. Next, it increased to 290 °C with a rate of 30 °C/min, and remained at 290 °C with a rate of 5 °C/min with a hold time of 3.5 min. After that, it increased to 310 °C with a rate of 30 °C/min and with a hold time of 4 min. Finally, the temperature increased to 319 °C. The temperature of the detector was set at 320 °C. The total program time was 35 min.

The fast evaporation of low molecular weight alkanes (from C_7_ to C_10_) and naphthalene prevented their inclusion in calibration curves. For the other hydrocarbons, calibration curves of the mix of alkanes (from C_11_ to C_30_) and the mix of the priority EPA-PAHs, excluding naphthalene but including 2-methylnaphthalene (2-MN), 1-methylnaphthalene (1-MN), were made using different concentrations of each hydrocarbon (1, 3, 5, 7 and 10 µg/mL) in triplicate. The standard deviation for the triplicate analyses of the target compounds was below 10%. The correlation coefficients (R^2^) were higher than 0.98 for all calibration curves.

### CO_2_ concentration measurement

To determine biodegradation activity of isolates, the CO_2_ concentration in the headspace of the serum bottles was measured with a Shimadzu Tracera GC-2010 Plus chromatograph fitted with a Carboxen 1010 (30 m × 0.58 mm × 30 μm) column with helium as carrier gas and a barrier ionization detector (BID). The injection size of each sample was 50 µL. The injector temperature was set at 250 °C to assure fast evaporation of the samples. The temperature of the column was initially programmed at 105 °C for a holding time of 7 min. After this, the temperature was incremented to 200 °C with a rate of 100 °C/min. The total program lasted 8 min. The calibration curve to measure CO_2_ was made using different volumes of air containing 600 ppm, 10,000 ppm, 20,000 ppm, 50,000 ppm, and 100,000 ppm of CO_2_ in triplicate. The coefficient of variation for the triplicate analyses of the target compounds was below 8%. The correlation coefficient of the calibration R^2^ was 0.997.

### Isolation of culturable strains

In order to isolate culturable bacterial strains, we performed serial dilutions of the soil incubation cultures and plated them on nutrient agar (NB; Nutrient broth No. 3, Sigma-Aldrich, 15 g/L agar, pH 7.4 at 30 °C) to support the growth of a wide range of soil bacteria. Colonies with different morphologies were further purified by restreaking on nutrient agar. The pure isolated strains were stored in Microbank™ vials according to the manufacturer’s instructions. After culturing the isolated bacteria in NB medium, their almost full-length 16 S rRNA gene sequences were amplified using the bacterial specific primers 8 F and 1512R (Weisburg et al. 1991; Felske et al. 1997). The resulting PCR products were sequenced bidirectionally by the Sanger method (1000 nt per read) (Macrogen Europe B.V.). Forward and reverse sequences were merged using the MEGA (v 7) software package. Sequences were compared online to sequences deposited in the nucleotide collection (nt) using megablast (default parameters) on the NCBI BLAST web site (Basic Local Alignment Search Tool, at www.ncbi.nim.nih.gov) (Zhang et al. 2000).

### DNA extraction and PCR amplification

Prior to DNA extraction, 50 ng of genomic DNA of *Geobacillus kaustophilus* (type strain ATCC 8005), which is a strain from the deep sea and unlikely to be found in soil, was added per gram of soil and milliliter of liquid media as an internal standard before 16 S rRNA gene sequencing. DNA was extracted with the ZymoBIOMICS DNA Miniprep Kit (Zymo Research, Irvine, CA, USA). For the DNA extraction, 250 mg of soil, 250 µL of media, and in the case of single strains, 300 µL of cells suspended in PBS were used. Samples were disrupted by shaking in a FastPrep 24 5G (California, USA) at 6.0 m/s for 60 s and subsequently processed according to the manufacturer’s protocols. To avoid cross-contamination of the samples, the process was performed with DNA-free equipment. The final elution of DNA was 100 µL in all cases. After extraction, DNA concentrations were measured by a Qubit 3.0 fluorometer (Invitrogen, Life technologies) using the Qubit dsDNA HS (high sensitivity) kit from Thermo Fisher Scientific. DNA samples were stored at -20 °C until further use. The PCR amplification (V3-V4 region) and sequencing on the Illumina MiSeq platform (Illumina, San Diego, USA) were done as previously described (Iturbe-Espinoza et al. 2021). The raw sequencing data were deposited in the BioProject database of the National Center for Biotechnology Information (NCBI) under accession number PRJNA701916.

### Data analysis

Sequencing reads were processed into an OTU table using USEARCH, as previously described (Persoon et al. 2017) with the following differences: after merging of the paired-end reads, and before clustering, all sequences were additionally quality-filtered using a maximum expected error rate of 0.005, no ambiguous bases allowed. Next, the subset of sequences with maximum expected error rate < 0.002 were clustered into OTUs using the default sequence similarity threshold of 97%. Finally, all sequences with passing the 0.005 threshold were mapped to the cluster centroids to produce the OTU table. For taxonomic assignments SILVA v 132 (Quast et al. 2013), was used. The SILVA sequences were trimmed to the V3-V4 16 S rRNA gene region as described previously (Koopman et al. 2016).

The dataset was subsampled to 15,500 reads per sample. The subsampled OTU table was used to estimate the total soil 16 S rRNA genes in each sample based on the relative abundance of the internal standard, the 16 S rRNA from *G. kaustophilus* (type strain ATCC 8005). We applied the formula proposed by (Smets et al. 2016) X$=\frac{Rs * W *C }{Ri *g}$, where X is the number of 16 S rRNA genes per sample, *Rs* is the number of reads of other taxa found in the soil (provided by the subsampled OTU table), *Ri* is the number of reads of *G. kaustophilus* (provided by the subsampled OTU table), *W* is the weight of the internal standard added to the sample (50 ng), *g* is the weight of the genome of *G. kaustophilus* (3.92993 × 10^−15^ g) (Daas et al. 2018)d is the number of 16 S rRNA gene copies in *G. kaustophilus*, assumed to be 10 (Stoddard et al. 2015). We recalculated the subsampled OTU table by multiplying the relative abundance of each OTU by the X*-*value computed for the corresponding sample.

The data was analyzed in R v 3.6.2 (R Core Team 2020) using phyloseq v 1.30.0 (McMurdie and Holmes 2013). The alpha-diversity indexes, OTU observed (which reflects the number of OTUs per sample) and Shannon diversity index (which considers the relative contribution of each OTU), were estimated using the recalculated OTU table based on the formula above that reflect the total soil 16 S rRNA genes in each sample. Additionally, a non-metric multidimensional scaling (NMDS) plot and heatmap plots were created to visualize patterns in beta-diversity. For differential abundance analyses (Log_2_-fold change), only OTUs with a 16 S rRNA gene number of more than or equal to 2,500,000, and occurring in 10% of samples, were selected. The abundance of the recalculated OTU table was normalized internally using a geometric mean that was calculated for each OTU across all samples in DESeq2 (v 1.26.0) (Love et al. 2014) using phyloseq. The *p* values were adjusted with the Benjamini and Hochberg correction method (Benjamini and Hochberg 1995) and an OTU was considered as differentially abundant if its mean proportion was significantly different between sample classes (*p* value < 0.01).

## Results

### Degradation of crude oil

Around one milligram of two types of soil with different degrees of oil contamination, one of them a less contaminated soil (L-soil) and the other a more contaminated soil (M-soil), were incubated in mineral media with crude oil spiked with PAHs and synthetic oil. The hydrocarbons were extracted in n-hexane from sacrificed cultures at the start of the experiment and after one, two, three, and six months of incubation, and quantified by GC-FID.

**Fig. 1:**
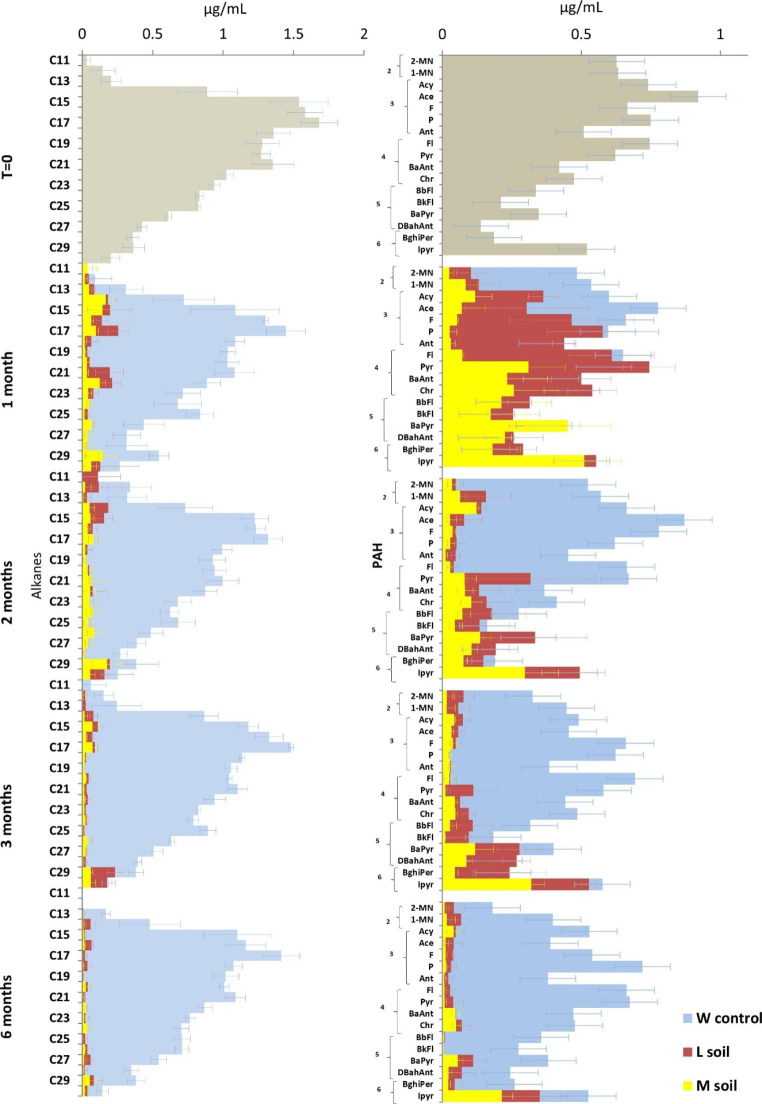
Quantification of specific hydrocarbons (C_11_-C_30_ alkanes and the sixteen priority PAHs according to the US-Environment Protection Agency (EPA)) in crude oil degraded by two soil communities: a less-contaminated soil (L-soil) and a more-contaminated soil (M-soil) compared with a weathering control without bacterial inoculum (W control). (**a**) Concentration of alkanes. (**b**) Concentration of PAHs. 2-methylnaphthalene (2-MN), 1-methylnaphthalene (1-MN), acenaphthylene (Acy), acenaphthene (Ace), fluorene (F), phenanthrene (P), anthracene (Ant), fluoranthene (Fl), pyrene (Pyr), benzo(a)anthracene (BaAnt), chrysene (Chr), benzo(b)fluoranthene (BbFl), benzo(k)fluoranthene (BkFl), benzo(a)pyrene (BaPyr), dibenz(a,h)anthracene (DBahAnt), benzo(ghi)perylene (BghiPer), and indeno(1,2,3-C,D)pyrene (Ipyr). Results are averages of biological triplicates with the error bars shown. The PAH subplots show the aromatic ring numbers

A relatively large fraction of hydrocarbons in the incubations of crude oil made part of an unresolved complex mixture (UCM) in the GC-chromatogram and was classified as unknown. Biodegradation is defined here as the percentual reduction of individual oil molecules. Both alkanes and PAHs were subjected to evaporation, especially the ones with a low molecular weight. The results showed that biodegradation of hydrocarbons was faster than their evaporation (Fig. 1). Based on the partial quantification of the identified hydrocarbons in the crude oil, we observed a reduction of hydrocarbons in both types of soil compared with their weathering controls which is inferred to be the result of biodegradation by microbial consortia (Fig. 1). Alkanes were degraded within one month, whereas the PAHs were degraded at rates dependent on the type of PAH. The consortium from the L-soil degraded more than 76% of the PAHs with two aromatic rings (2-methylnaphthalene (2-MN) and 1-methylnaphthalene (1-MN) within one month. During the same period, the consortium from the M-soil degraded the same types of PAHs, but also those with three aromatic rings and fluoranthene (Fl), which has four aromatic rings. After two months, the consortium from the L-soil degraded a large fraction of all the PAHs with three aromatic rings and fluoranthene, along with a degradation of 53% of PAHs with four aromatic rings. The consortium from the M-soil performed much better as it additionally degraded 72% of benzo(b)fluoranthene (BbFl) and benzo(k)fluoranthene (BkFl), which have five aromatic rings. After three months, the consortium from the L-soil had degraded 66% of the PAHs with four and five aromatic rings, while the consortium from the M-soil degraded additionally PAHs with five and six aromatic rings. After six months, both consortia had degraded the majority of PAHs with six aromatic rings except for indeno(1,2,3-C,D)pyrene (Ipyr), which was hardly degraded by the consortium from the L-soil but for around 60% by the consortium from the M-soil.

**Fig. 2:**
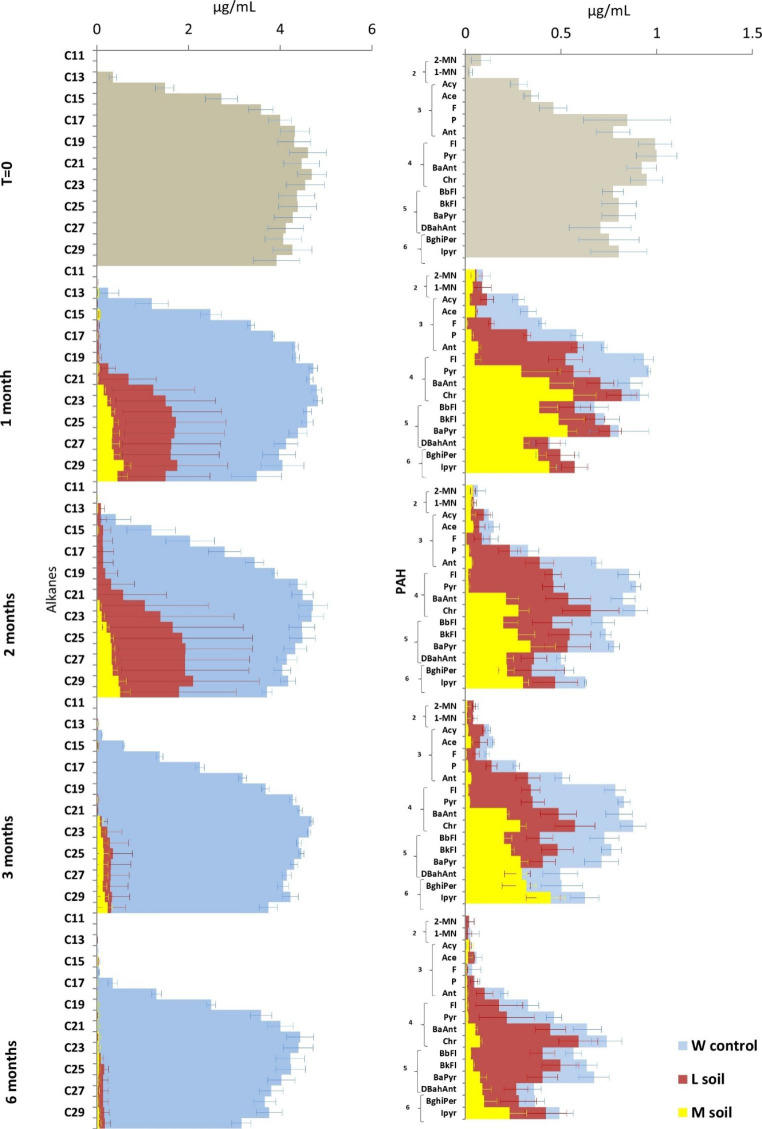
Quantification of synthetic oil molecules degraded by two soil communities, one from a less-contaminated soil (L-soil) and one from a more-contaminated soil (M-soil) compared with a weathering control without bacterial inoculum (W control). (**a**) Concentration of alkanes (C_11_-C_30_). (**b**) Concentration of the sixteen priority PAHs according to the US-Environment Protection Agency (EPA). 2-methylnaphthalene (2-MN), 1-methylnaphthalene (1-MN), acenaphthylene (Acy), acenaphthene (Ace), fluorene (F), phenanthrene (P), anthracene (Ant), fluoranthene (Fl), pyrene (Pyr), benzo(a)anthracene (BaAnt), chrysene (Chr), benzo(b)fluoranthene (BbFl), benzo(k)fluoranthene (BkFl), benzo(a)pyrene (BaPyr), dibenz(a,h)anthracene (DBahAnt), benzo(ghi)perylene (BghiPer), and indeno(1,2,3-C,D)pyrene (Ipyr). Results are averages of biological triplicate with the error bars shown. The PAH subplots show the aromatic ring numbers

In the incubations with synthetic oil, both consortia also biodegraded the hydrocarbons while loss of the more volatile molecules was attributed to evaporation (Fig. 2). The consortium from the M-soil degraded up to 95% of alkanes within one month while the consortium from the L-soil did the same within three months. The degradation of PAHs was more heterogeneous. Within one month, the consortium from the L-soil degraded mainly the 37% of light PAHs with two aromatic rings (2-methylnaphthalene (2-MN) and 1-methylnaphthalene (1-MN)) while the consortium from the M-soil also degraded 92% of PAHs with three aromatic rings and 94% of fluoranthene (Fl), which contains four aromatic rings. After two months, the consortium from the L-soil succeeded in the degradation of 36% of PAHs with three aromatic rings while the consortium from the M-soil already degraded a 98% of pyrene (Pyr), which also contains four aromatic rings. The degradation patterns after three months of cultivation did not deviate so much from those after two months of incubation, regardless of the soil type. After six months of cultivation, no further degradation of PAHs was observed by the consortium from the L-soil, while the consortium from the M-soil biodegraded a relatively larger part of the molecules with six aromatic rings, such as the 73% of benzo(ghi)perylene and 53% of indeno(1,2,3-C,D)pyrene.

Based on the biodegradation results during the sampling times shown in Figs. 1 and 2, we concluded that the microbial community from the more-contaminated soil had a higher hydrocarbon biodegradation capacity than the one from the less-contaminated soil in cultures both with crude oil (unpaired t-test, *p*  < 0.05 at 6 months of incubation) and with synthetic oil (unpaired t-test, *p*  < 0.01 at 6 months of incubation).

### Community dynamics

**Fig. 3:**
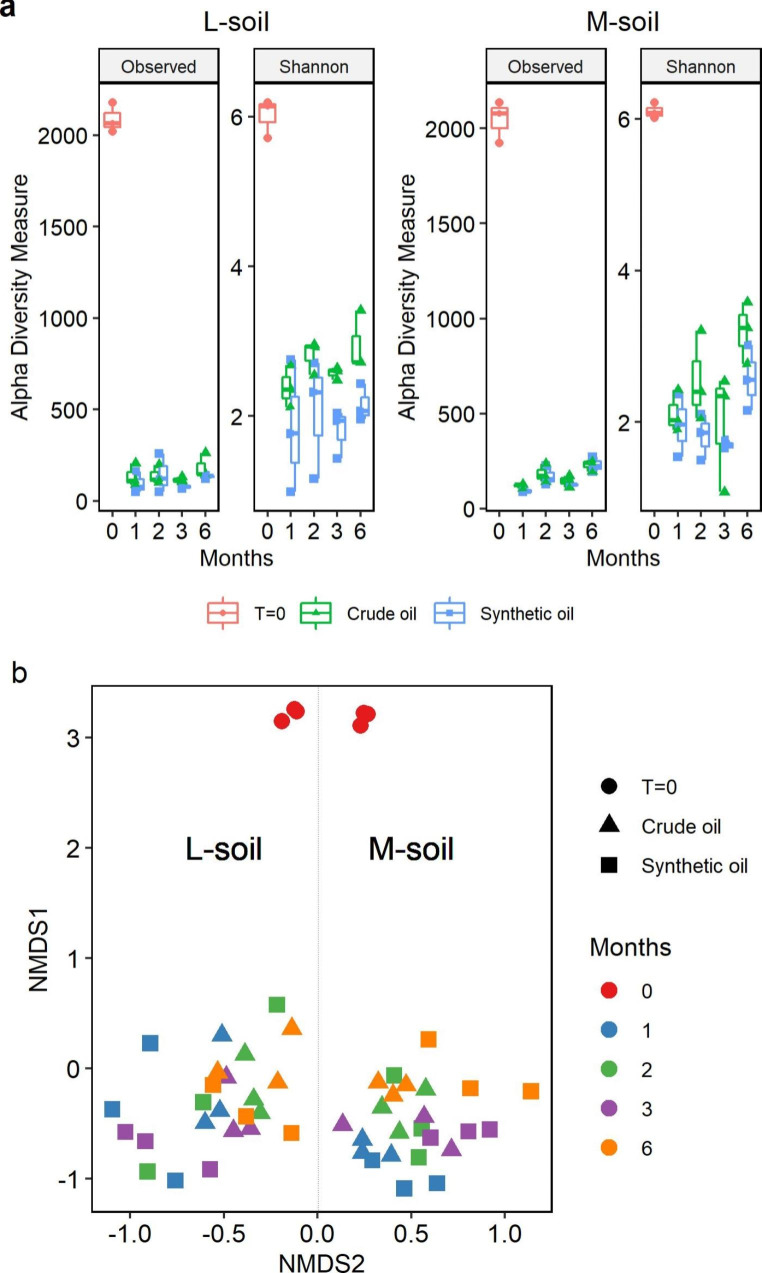
Diversity of two oil-contaminated soils incubated in mineral media with crude oil (spiked with EPA-PAHs) or a synthetic oil. L-soil is a less contaminated soil and M-soil is a more contaminated soil. (**a**) Alpha-diversity indices. Observed index represents the total number of OTUs per sample. Shannon index represents the relative abundance of each OTU. (**b**) Non-metric multidimensional scaling (NMDS) ordination of the Bray-Curtiss dissimilarity between communities. All NMDS2 values of communities from the L-soil were negative while the same values were positive in case of communities from the M-soil. A dotted line was added to separate the clustering affinity of the bacterial communities from both types of soil

16 S rRNA gene amplicon sequencing was performed to reveal the succession of the microbial community during the degradation of crude oil and synthetic oil. The alpha diversity indices, Shannon and OTU observed, are shown in Fig. 3a. 2088 and 2045 OTUs were observed at the start of the incubation suggestive for a high initial biodiversity in both L-soil and M-soil, respectively. After one month of incubation and the subsequent sampling times, there was a reduction of richness until below 500 OTUs. Likewise, the Shannon index also decreased from around six at the start of the incubation to less than four at the next sampling times. Communities degrading the crude oil were more diverse than communities degrading the synthetic oil regardless of the types of soil used for the inoculation. Those changes in local diversity were also seen in a non-metric multidimensional scaling (NMDS) plot of Bray–Curtiss dissimilarity (Fig. 3b). This plot indicated an evident differentiation between the community structures of the two types of soil but not a clear differentiation between crude oil and synthetic oil or between sampling times after the start of the experiment.

In order to understand which types of organisms may be involved in hydrocarbon biodegradation, we compared the heatmap of the OTU abundances with the differential analyses (log2-fold change) that represent each taxon whose abundance significantly changed (*p* < 0.01) from the original inoculum (T = 0) versus the subsequent sampling times (Supplementary Fig. 2). The community profiles of the biological triplicates were similar at the start of the experiment, but they started to deviate from one another during prolonged cultivation. Despite the deviating responses of the triplicates, certain genera increased in abundances both in crude oil and in synthetic oil as judged by their log_2_-fold changes.

The abundance of members of the genera *Sphingopyxis, Aeromonas, Rhizobium, Pseudoxanthomonas, Mycobacterium, Hydrogenophaga, Dyela* and *Stenothrophobacter* increased in a range from 5 to 30 Log_2_ fold change during the incubation of L-soil, regardless of the type of oil. There were also differences depending on the type of oil. A species from the genus *Halothiobacillus*, a sulfur-oxidizing bacterium, was prevalent in the incubation with crude oil but not in the incubation with synthetic oil, which lacks sulfur containing components. In the case of the M-soil, the abundance of members of the genera *Stenothrophomonas, Pseudomonas, Rhodanobacter, Sediminibacterium*, and *Novosphingobium* increased during incubation in a range from 2 to 30 Log_2_ fold change, regardless of the type of oil. In this experiment we noticed differences depending on the type of oil as well. A species from the genus *Sulfuritalea*, another sulfur-oxidizing bacterium, was prevalent in the incubation with crude oil but not in those with synthetic oil.

### Hydrocarbon degradation linked to the succession of microbial communities

**Fig. 4:**
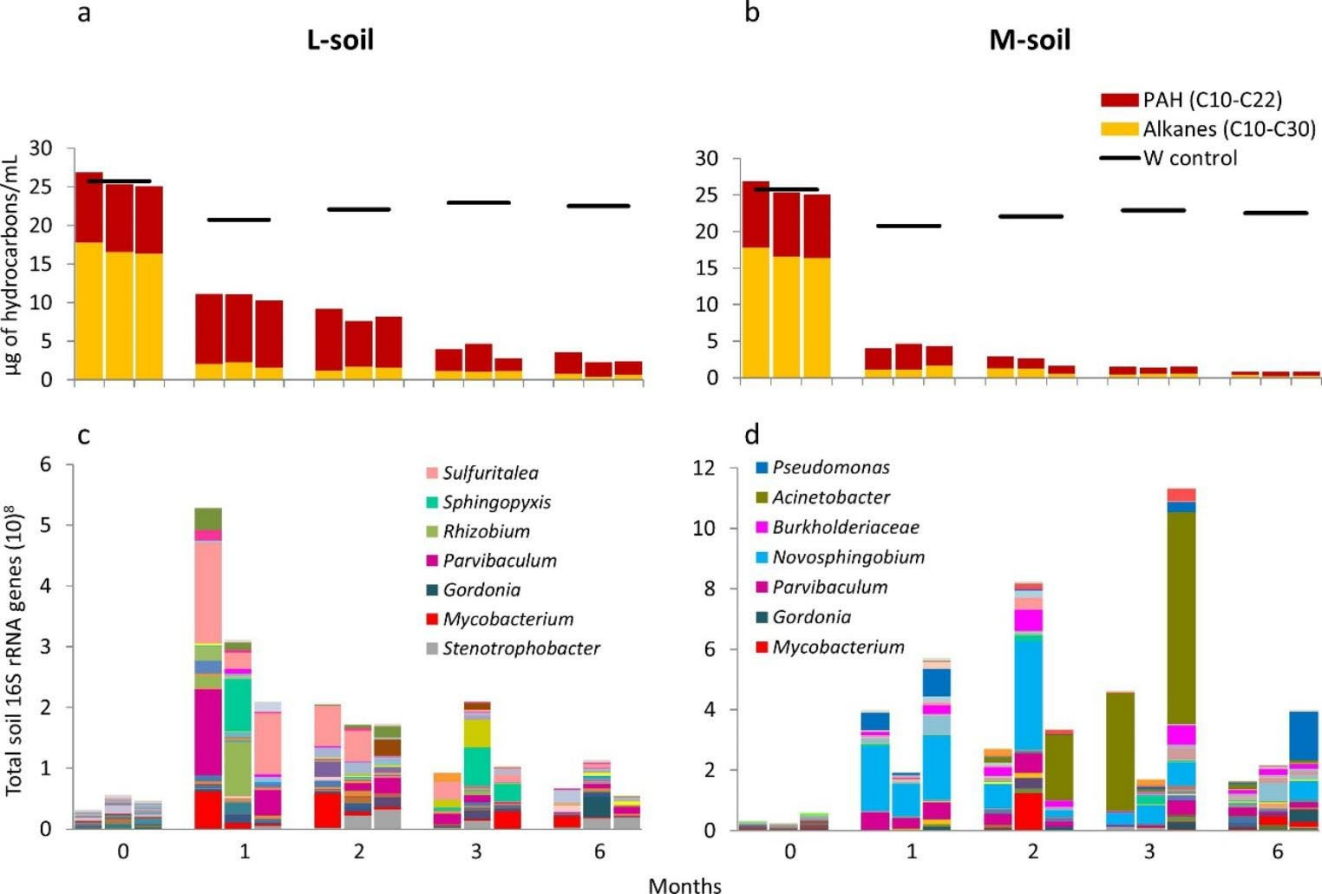
Quantification of specific hydrocarbons (C_11_-C_30_ alkanes and the sixteen priority PAH according to the US-Environment Protection Agency (EPA)) linked to community changes. The crude oil was degraded by two soils: one less-contaminated (L-soil) and another more-contaminated (M-soil) in comparison with a weathering control without bacterial inoculum (W control). The summation of alkanes and the summation of PAHs (**a** and **b**) are affiliated with the bar plots of the abundance of the microbial community composition at the genus level (**c** and **d**). Only the most abundant genera (the top 7) or family level (in cases of unclassified genera) are listed in the legends

**Fig. 5:**
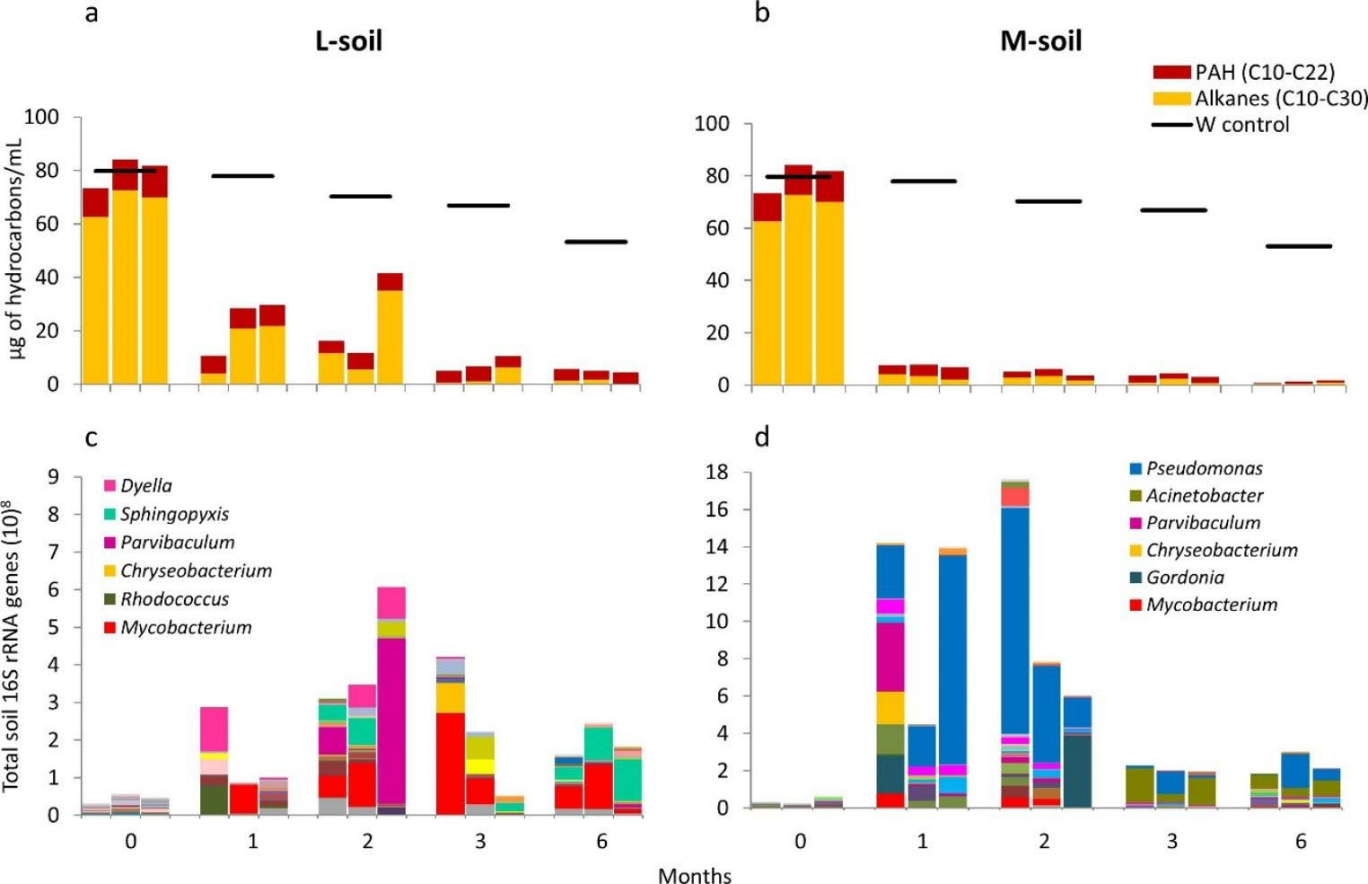
Quantification of hydrocarbons in a synthetic oil linked to community changes. The synthetic oil was degraded by two soils: one less-contaminated (L-soil) and another more-contaminated (M-soil) in comparison with a weathering control without bacterial inoculum (W control). The summation of alkanes (C_11_-C_30_) and the summation of the sixteen priority PAH according to the US-Environment Protection Agency (EPA) (**a** and **b**) are affiliated with the bar plots of the abundance of the microbial community composition at the genus level (**c** and **d**). Only the most abundant genera (the top 6) are listed in the legends

To improve our understanding of the succession of the microbial community involved in biodegradation of hydrocarbons, bar plots of the genera in the community profiles indicating the abundance of the 16 S rRNA gene were correlated to the total concentration of hydrocarbons (quantified by GC-FID) in µg of hydrocarbon/mL (Figs. 4 and 5). At the start of the experiment, one mg of soil contained less than 10^8^ 16 S rRNA genes under all experimental conditions inferred from the DNA internal standard. At this sampling time, the serum bottles contained 25 µg of hydrocarbons/mL from the crude oil spiked with EPA-PAHs (specific quantification) or 80 µg of hydrocarbons/mL from the synthetic oil (total quantification).

The biological triplicates showed varying community profiles in the incubations with crude oil (Fig. 4). The degradation of the n-alkanes by bacterial communities from both types of soil within the first month correlated with an increase in the community abundance, estimated from the total soil 16 S rRNA gene copy number, by approximately a factor of 10. During this time, the consortia from the L-soil degraded less than 10% of PAHs while the consortia from the M-soil degraded more than 50% of PAHs. Between one and six months, the community size of the L-soil decreased in time while the biodegradation of PAHs was less efficient and slower compared to the one observed from the M-soil. In contrast to the L-soil, the community size of the M-soil increased during three months of incubation, which may be the result of degradation of PAHs. After three months of incubation, the size of both communities decreased. During cultivation of the L-soil, there were a few genera that were present multiple times, such as *Sulfuritalea, Mycobacterium*, and *Sphingopyxis*. In consortia from the M-soil, members of genus *Novosphingobium* were dominant after one and two months, and members of *Acinetobacter* between two and three months. Although we observed a clear dominance of these genera, the succession of the microbial communities was apparently variable between the biological triplicates in both types of soil incubated with crude oil.

The communities of the biological triplicates of the incubations with the synthetic oil were different in most of the cases, but more similar compared to the profiles of the degradation of the crude oil (Fig. 5). The highest values for the total soil 16 S rRNA genes were reached after two months of cultivation of both types of soil. The consortia from the L-soil did not degraded the n-alkanes entirely within two months. After three and six months of incubation, the sizes of both communities decreased, perhaps due to the absence of suitable carbon and energy sources or by the accumulation of toxic intermediates. After 6 months, the degradation of PAHs by the consortia from the L-soil was still incomplete while it was more effective by the consortia from the M-soil. Regarding the genus composition in the community profiles, we observed a clear dominance of members of the genera *Mycobacterium* and *Sphingopyxis* in consortia from the L-soil and members of the genera *Pseudomonas* and *Acinetobacter* in the consortia from the M-soil.

### Isolation and identification of bacterial species associated to oil degradation

The succession of the microbial community was further investigated by counting colony-forming units (CFUs), and isolation of dominant colonies by plating on nutrient agar plates. We isolated and sequenced the 16 S rRNA genes of 31 unique bacterial colonies from both types of soil during the whole experiment. Table 1 shows the tentative names of the different isolates based on their closest matches and the new accession numbers of the 16 S rRNA gene sequences deposited in GenBank.

**Table 1:** List of bacterial species isolated during incubation of the different types of soil with crude oil (oil) or synthetic oil (mix) in mineral media

Tentative names	Coverage	% Identity	Soil type	Carbon source	Isolation time (months)	Accession number
*Gordonia sp.* PT0S1a	100%	100%	L	oil, mix	0	MZ427249
*Methylorubrum suomiense* PT0S1b	100%	100%	L	oil, mix	0	MZ427250
*Rhodococcus sp.* P1MC1M3	99%	100%	L	mix	1	MZ427251
*Achromobacter sp.* P1MC1M1	100%	100%	L	mix	1	MZ427260
*Microbacterium oxydans* P1MC1O1	100%	100%	L	oil	1	MZ427252
*Sphingopyxis sp.* P1MC1O2	100%	100%	L	oil	1	MZ427253
*Thermomonas sp.* P1MC1O3	100%	100%	L	oil, mix	1, 6	MZ427254
*Bosea sp.* P2MC1M1a	100%	100%	L	oil, mix	2, 6	MZ427255
*Sphingomonas sp.* P2MC1M1b	100%	100%	L	mix	2, 3	MZ427256
*Bacillus sp.* P2MC1M2	100%	100%	L	mix	2	MZ427257
*Dyella sp.* P2MC1M3	100%	100%	L	oil, mix	2	MZ427258
*Pseudoxanthomonas sp.* P2MC1O3	100%	100%	L	oil	2	MZ427259
*Xanthobacter sp* P2MC1O1	100%	100%	L	oil	2	MZ427261
*Hydrogenophaga taeniospiralis* P3MC1M2	100%	100%	L	mix	2	MZ427262
*Stenotrophomonas acidaminiphila* P3MC1M3	100%	100%	L	oil, mix	3	MZ427263
*Micromonospora aurantiaca* P6MC1M3	100%	100%	L	oil	6	MZ427264
*Bacillus sp.* PT0S19a	100%	100%	M	oil, mix	0	MZ427265
*Hydrogenophaga taeniospiralis* PT0S19b	100%	100%	M	oil, mix	0	MZ427266
*Rhodococcus sp.* PT0S19c	100%	100%	M	oil, mix	0	MZ427267
*Gordonia amicalis* P1MC4M1	100%	100%	M	mix	1, 2	MZ427269
*Stenotrophomonas acidaminiphila* P1MC4M3	100%	100%	M	mix	1	MZ427270
*Sphingopyxis sp.* P1MC4O1	100%	100%	M	oil	1	MZ427268
*Rhodanobacter lindaniclasticus* P1MC4O1a	99%	100%	M	oil	2	MZ427271
*Bosea sp.* P1MC4O1b	100%	100%	M	oil, mix	1, 2, 3, 6	MZ427272
*Sphingomonas sp.* P1MC4O3a	100%	100%	M	oil, mix	1, 3, 6	MZ427273
*Chryseobacterium koreense* P1MC4O3b	99%	100%	M	oil	1, 3, 6	MZ427274
*Ensifer adhaerens* P3MC4M1a	100%	100%	M	mix	3	MZ427275
*Novosphingobium flavum* P3MC4M1b	99%	100%	M	mix	3	MZ427276
*Pseudoxanthomonas sp.* P3MC4M3	99%	100%	M	mix	1, 2, 3, 6	MZ427277
*Pseudomonas sp.* P3MC4O1	100%	100%	M	oil, mix	3	MZ427278
*Rhizobium daejeonense* P6MC4O1	99%	100%	M	oil	6	MZ427279

The first column shows the tentative taxonomic assignment of the isolates based on the closest hit after a MEGABLAST search. The second column is the percentage of coverage. The third column shows the type of oil-contaminated soil: less contaminated (L) and more contaminated (M). The fourth column shows the carbon source used in the incubation: crude oil (‘oil’; spiked with EPA-PAHs) or synthetic oil (‘mix’; a mixture of alkanes (C_11_-C_30_) and EPA-PAHs). The fifth column indicates the time of cultivation at which the species were isolated. The sixth column shows the accession numbers in GenBank of the new 16 S rRNA gene sequences

We tracked the relative abundance of genera of the community profiles resulting from the 16 S rRNA gene amplicon sequencing data. This data is presented in the Supplementary Tables 1 and 2. Most of the bacteria isolated on nutrient agar were not representative of the dominant bacterial OTUs recovered by Illumina sequencing considering that most soil bacteria are not cultivable in the laboratory. However, there were a few exceptions: members of the genera *Dyela* (10.7%), *Rhodococcus* (11.1%), *Sphingomonas* (7.9%) and *Sphingopyxis* (39%) from the L-soil, *Gordonia* (21.1%) and *Pseudomonas* (53.8%) from the M-soil appeared with high numbers of colonies on the plates and had also a high relative contribution in the community profiles (values in between brackets).

### Hydrocarbon biodegradation by single isolates

Based on the rate growth of pre-cultures in nutrient broth, we selected 25 of the 31 isolates to test if they were able to biodegrade hydrocarbons in pure bacterial species cultures, each of them representing a different unique genus. The incubations were performed in duplicate using the synthetic oil described in Material and Methods. Media with hydrocarbons but without a bacterial inoculum were used as weathering controls (W control). The increase of the CO_2_ concentration in the headspace indicated biodegradation of hydrocarbons in the serum bottles. After one month and six months of incubation, we observed the production of CO_2_ by *Gordonia sp.* PT0S1a, *Micromonospora aurantiaca* P6MC1M3, *Microbacterium oxydans* P1MC1O1, *Rhodococcus sp.* P1MC1M3, *Gordonia amicalis* P1MC4M1, and *Pseudomonas sp.* P3MC4O1 as shown in the Supplementary Fig. 4. Subsequently, we performed a hydrocarbon extraction in n-hexane for further GC-FID quantification of the cultures of those isolates after six months of incubation.

**Fig. 6:**
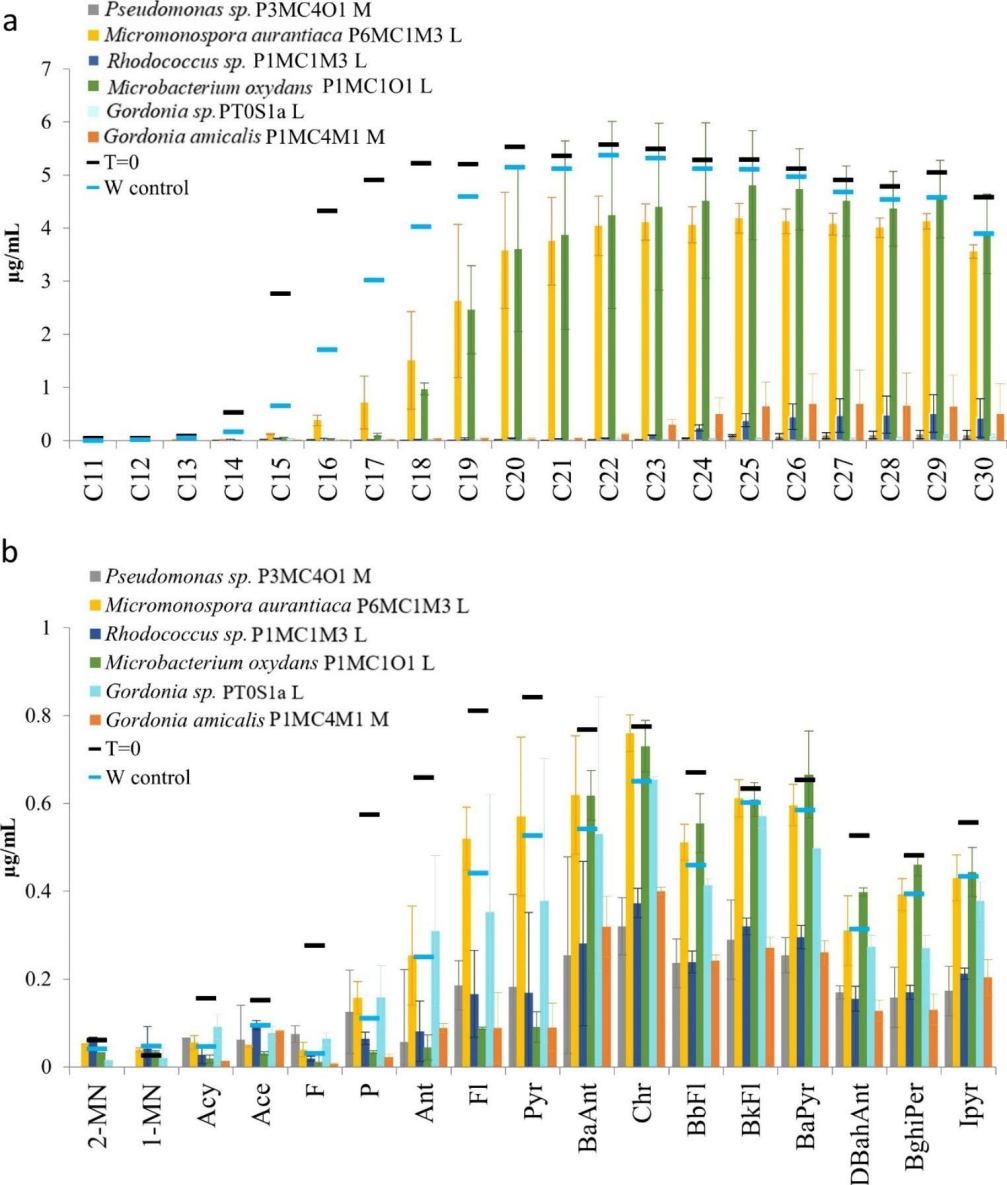
Degradation of a synthetic oil by single species in isolation. Cultures were made using 10 mL of medium in 30 mL glass bottles closed with Teflon caps. Incubations were at 30 °C, shaking at 120 rpm for six months. The weathering control (W control) did not contain a bacterial inoculum. (**a**) Degradation of n-alkanes (C_11_ to C_30_). (**b**) Degradation of PAHs. 2-methylnaphthalene (2-MN), 1-methylnaphthalene (1-MN), acenaphthylene (Acy), acenaphthene (Ace), fluorene (F), phenanthrene (P), anthracene (Ant), fluoranthene (Fl), pyrene (Pyr), benzo(a)anthracene (BaAnt), chrysene (Chr), benzo(b)fluoranthene (BbFl), benzo(k)fluoranthene (BkFl), benzo(a)pyrene (BaPyr), dibenz(a,h)anthracene (DBahAnt), benzo(ghi)perylene (BghiPer), and indeno(1,2,3-C,D)pyrene (Ipyr). Results are averages of biological triplicate with the error bars shown

The biodegradation of single hydrocarbons by isolates is illustrated in Fig. 6. The four isolates with the highest CO_2_ production, *Rhodococcus sp.* P1MC1M3, *Pseudomonas sp*. P3MC4O1, *Gordonia sp*. PT0S1a and *Gordonia amicalis* P1MC4M1, degraded almost completely the n-alkanes during six months of incubation (Fig. 6a). In contrast, the other two strains with less CO_2_ production, *Microbacterium oxydans* P1MC1O1 *and Micromonospora aurantiaca* P6MC1M3 only partially degraded the n-alkanes during six months of incubation.

Although the amount of each PAH added in the media was five times less than the amount of each alkane, the degradation of PAHs by all six strains was slower and incomplete (Fig. 6b). Members of the species *Microbacterium oxydans* P1MC1O1, *Micromonospora aurantiaca* P6MC1M3 and *Gordonia sp.* PT0S1a did not degrade PAHs since the PAHs concentration was similar compared to the weathering control. *Gordonia amicalis* P1MC4M1, *Pseudomonas sp.* P3MC4O1, and *Rhodococcus sp.* P1MC1M3 performed better with the degradation of the PAHs. These last three species degraded all the two and three aromatic ring PAHs, a large part of four aromatic ring PAHs such as fluoranthene and pyrene, and around 50% of the other PAHs with four, five and six aromatic rings in their structure.

## Discussion

In this study, we determined the hydrocarbon biodegradation capacity of microbial communities using batch liquid systems inoculated with only one milligram of soil from two different origins, each with a different extent of contamination with petroleum hydrocarbons. Bacterial cell densities in soil range from ca. 10^7^ to 10^10^ cells per gram of soil (Bickel and Or 2020). Assuming a bacterial density of 10^8^ cells per gram of soil, which is a commonly reported value, there would be approximately 10^5^ bacterial cells in one milligram of soil.

Our results show that hydrocarbon biodegradation occurs in the contaminated soils from the shoreline cleanup and assessment project (Bonte et al. 2020). The contrast in hydrocarbon biodegradation between natural and synthetic oil in the incubations is remarkable: the biodegradation of hydrocarbons in synthetic oil was slower than in natural oil. This is an important finding in the context of risk management as environmental organizations over the world want to reduce the reliance on fossil fuels while the use of synthetic fuels is a growing trend (Mouradian et al. 2023).

The microbial communities present in the two types of soil were able to degrade hydrocarbons that we added to the cultures, but the one from the less-contaminated soil performed less efficient than the one from the more-contaminated soil. The likely explanation is that pre-exposure of the microbial community to the higher concentrations of hydrocarbons enhances the oil degradation rate. Indeed, hydrocarbon contamination is one of several factors that determines the capacity to degrade hydrocarbons (Morales-Guzmán et al. 2017) as well as xenobiotic contaminants (Kim et al. 2017). Pre-exposure to xenobiotics in general usually results in adaptation of the microbial community towards a better degradation of these contaminants (Poursat et al. 2019). The results of this study confirmed earlier findings that individual components of the oil are degraded at different rates, with the highest rates observed for n-alkanes, followed by low molecular weight aromatics and ultimately by the more complex PAHs (Head et al. 2006; Röling and van Bodegom 2014).

Both soil communities showed a reduction in the alpha diversity indices between the start of cultivation and after one month. This reduction may in part be caused by the dilution effect of inoculating one milligram of soil in the ten milliliters of media. Another major driver is likely the metabolic potential of a limited set of species from the community to use the hydrocarbons or their metabolic breakdown products as carbon and energy source. The difference in adaptability between the two types of soil suggests that oil-degrading bacteria were present in a higher proportion in the more-contaminated soil compared to the less-contaminated soil resulting in faster degradation of the hydrocarbons by the consortia from the more-contaminated soil.

The community profiles of soil incubated with crude oil were more variable than those incubated with synthetic oil. This variability can be explained by the more diverse hydrocarbon composition of the crude oil. Each of the more than hundreds of types of oil molecules is a potential carbon substrate that may cause competition among community members with similar genetic capabilities, resulting in more heterogeneous community profiles than the ones incubated with synthetic oil. Despite starting with the same conditions and inocula, the stochastic community assembly of different competitive endpoints can also be explained by the initial complexity of both soil communities (with OTU richness over 2000). The inoculum communities between the different starting amounts of soil added to each batch culture could give slightly different starting points. These slight differences can promote differences in subsequent community composition by the alteration of priority effects referred to as the timing of the arrival of bacterial species within the microbial community succession (Weidlich et al. 2021; Debray et al. 2022). Another potential contribution factor is the presence of PCR inhibitors in the crude oil and in the contaminated soils which could also interfere with the DNA amplification and increase the variability in community structure and size (Puentes et al. 2019; Basim et al. 2020).

The community size of the less-contaminated soil was relatively high during the first month of incubation when more readily biodegradable compounds such as alkanes were consumed, but once they were depleted, the community size decreased again. In contrast, the community size of the more contaminated soil increased after the depletion of alkanes, which we believe is the result of degradation of PAHs that were then used as additional carbon and energy sources. Interestingly, there was a clear dominance of only two genera per soil type at the last sampling times in the incubations with synthetic oil, indicating that a more stable bacterial consortium with very few bacterial species developed during the degradation of complex PAHs. Members of the genera *Sphingopyxis* and *Mycobacterium* were prevalent in the less-contaminated soil (Fig. 5c), which was also reported previously in PAH-contaminated mangroves (Guo et al. 2010, 2011). Members of the genera *Acinetobacter* and *Pseudomonas* were prevalent in the more-contaminated soil (Fig. 5d). Similar species were isolated from oil-contaminated soil worldwide and were shown to produce biosurfactants and to biodegrade hydrocarbons (Sugimori and Utsue 2012; Parthipan et al. 2017; Méndez et al. 2017).

Another striking difference between the incubations was the increase in abundances of sulfur-oxidizing bacteria in crude oil incubations such as members of the genera *Halothiobacillus* and *Sulfuritalea* indicating their potential role in biodegrading sulfur components (Li et al. 2014; Sperfeld et al. 2019; Rodrigue et al. 2020; Wei et al. 2020) whose concentration in the Bonny crude oil is from 0.1 to 0.2% (Nuhu et al. 2022). Biodegradation of both alkanes and PAHs in the synthetic oil was slower than in the natural oil. This finding is interesting in the context of the sulfur-specific bacteria which were observed suggesting that sulfur and perhaps other trace elements present in natural crude are important for bacterial communities that can degrade PAHs.

We also isolated 31 unique bacterial species from our cultures that appeared as dominant colonies on nutrient agar plates. Most of them did not correlate with OTUs that had a high abundance in the amplicon-based community profiles as suggested in other studies (Stefani et al. 2015) due to the fact that most bacteria are difficult to culture in isolation. We identified only six hydrocarbon-degrading specialists from the 25 species tested. All of them had the metabolic capacity to degrade alkanes from C_11_ to C_30_ as judged by the production of CO_2_ in the headspace of the isolated species (Supplementary Fig. 4). Additionally, the members of the genera *Pseudomonas*, *Gordonia* and *Rhodococcus* were able to degrade high molecular weight PAHs during six months of incubation. Other studies reported that those three genera are indeed degraders of PAHs (Pizzul et al. 2006; Song et al. 2011; Isaac et al. 2015). The two species of *Gordonia* had different biodegradation capacities. *Gordonia* isolated from the less contaminated soil did not degrade complex PAHs, but the one isolated from the more-contaminated soil did. We isolated two species, presumably with different genomes affecting their biodegradation capacities. Further genome sequencing is necessary to address this hypothesis.

Some of our isolates reported as oil-degrading specialists did not degrade hydrocarbons. This was the case for sphingomonads, which includes the genera *Sphingomonas*, *Novosphingobium* and *Sphingopyxis* (Maeda et al. 2020). Species of these genera were isolated on plate whereas their relative contribution to the community profiles was 8%, 2% and 39%, respectively. We believe that the mineral media that we provided were not optimal to stimulate hydrocarbon biodegradation by those species. Another explanation would be that sphingomonads need to interact with other community members such as surfactant producers to initiate hydrocarbon biodegradation. Future experiments are, therefore, aimed at repeating the biodegradation assays using different media compositions and different incubation conditions to unravel their metabolic potential and possible interactions.

In conclusion, the experiments showed varying biodegradation rates of both alkanes and PAHs between natural and synthetic oil with relatively more efficient biodegradation in natural crude oil. Based on the presence of species from the genera *Sulfuritalea* and *Halothiobacillus*, sulfur-oxidizing bacteria in the incubation with crude oil, we hypothesize that the difference in biodegradation may be due to the presence of sulfur in natural oil while these sulfur species are absent in synthetic oil. Oil-degrading bacteria compete for the most readily biodegradable compounds while the size of the bacterial community increases and is more variable in composition. This stochastic community assembly may be the result of the complexity of crude oil composition, initial biodiversity, and the size of the community inocula. In contrast, during the degradation of most complex PAHs, the community size decrease, and the microbial composition is more homogeneous. The isolates *Gordonia* sp. PT0S1, *Micromonospora aurantiaca* P6MC1M3, *Microbacterium oxydans* P1MC1O1, *Rhodococcus sp.* P1MC1M3, *Gordonia amicalis* P1MC4M1, and *Pseudomonas sp*. P3MC4O1 are candidates for initial oil biodegradation. Ultimately, our better understanding of the microbial consortia assembly during oil degradation and the identification of key players in that process may well assist in improving future bioremediation efforts, for example by the inoculation or bioaugmentation of primary oil degraders during ex-situ biodegradation such as biodigesters or landfarming.

## Electronic supplementary material

Below is the link to the electronic supplementary material.

Supplementary Material 1

## Data Availability

All data generated or analyzed during this study are included in this article or at the public database of NCBI with accession numbers presented.
