# Supplementary material for: Correlating the succession of microbial communities from Nigerian soils to petroleum biodegradation
Source: World J Microbiol Biotechnol. 2023 Jul 1;39(9):239. doi: 10.1007/s11274-023-03656-7 (PMC10314880; doi:10.1007/s11274-023-03656-7)
Supplement: Supplementary file 1 — Supplementary Material 1 [file 11274_2023_3656_MOESM1_ESM.docx]

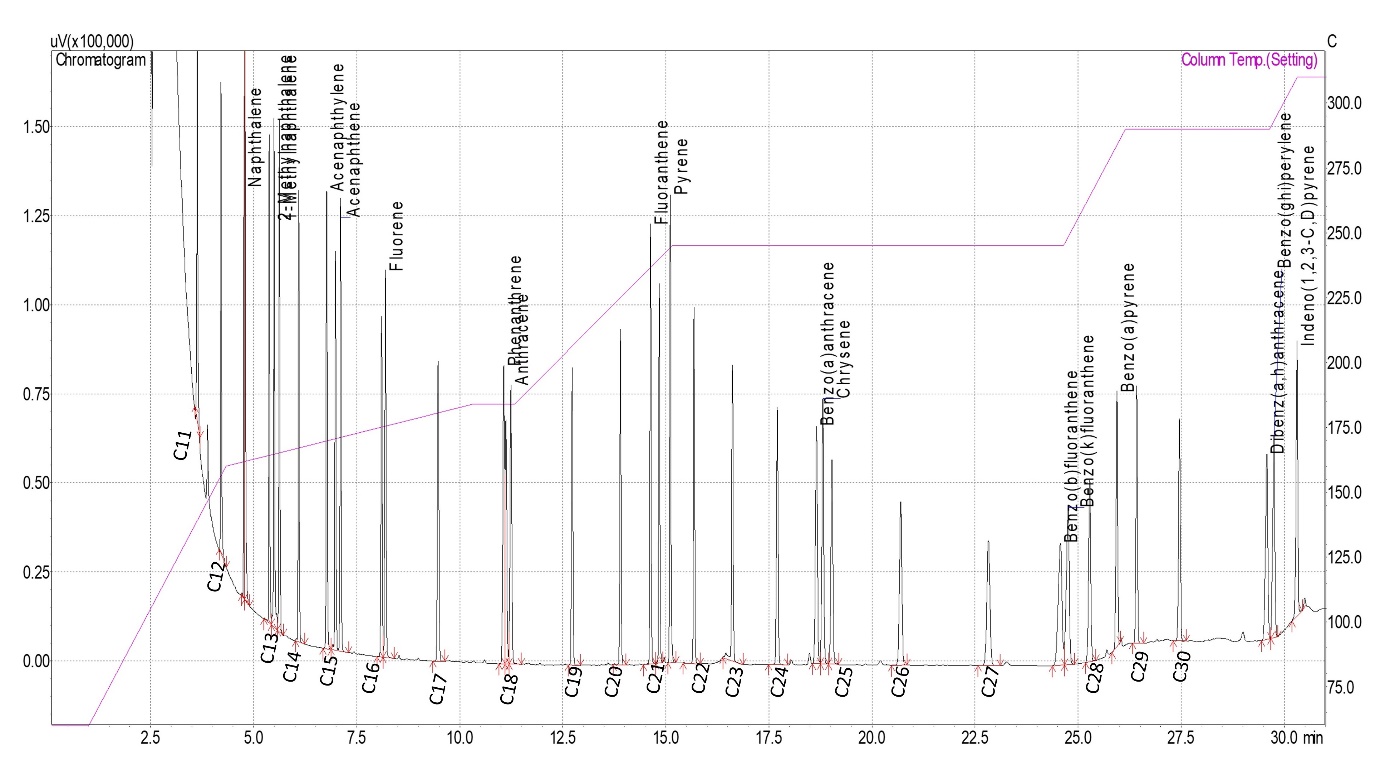


**Supplementary Figure 1.** Chromatogram of GC-FID analysis of n-alkanes and PAHs without fractionation.


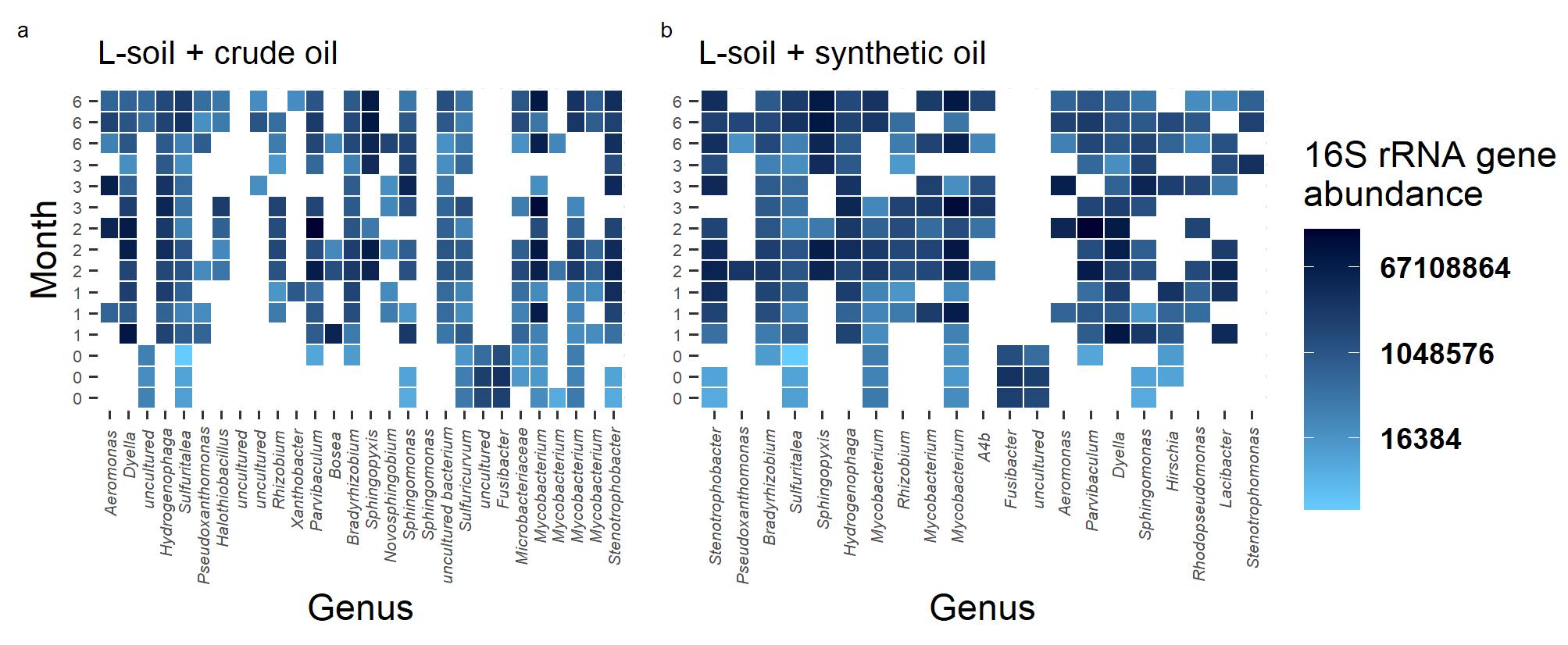

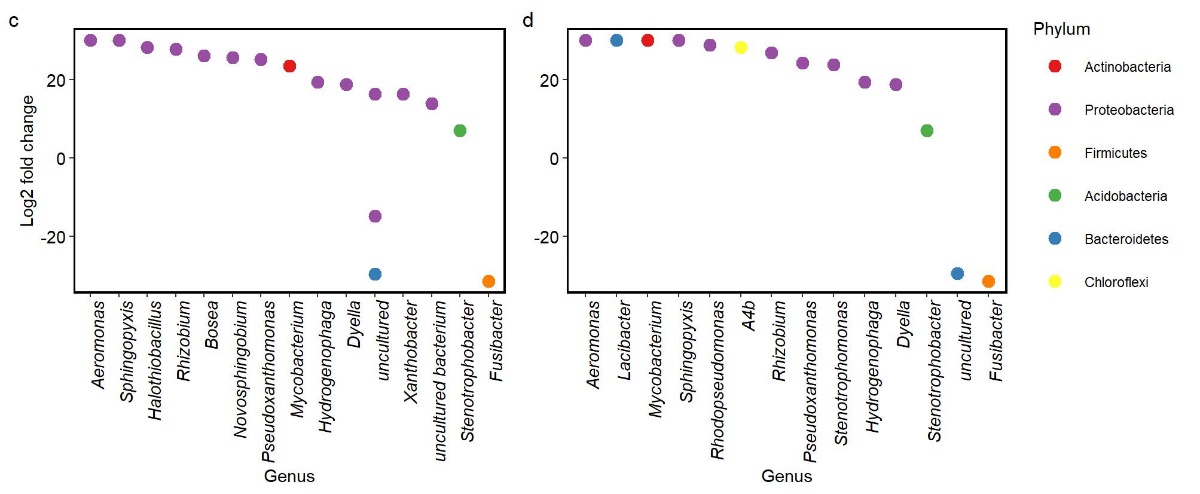

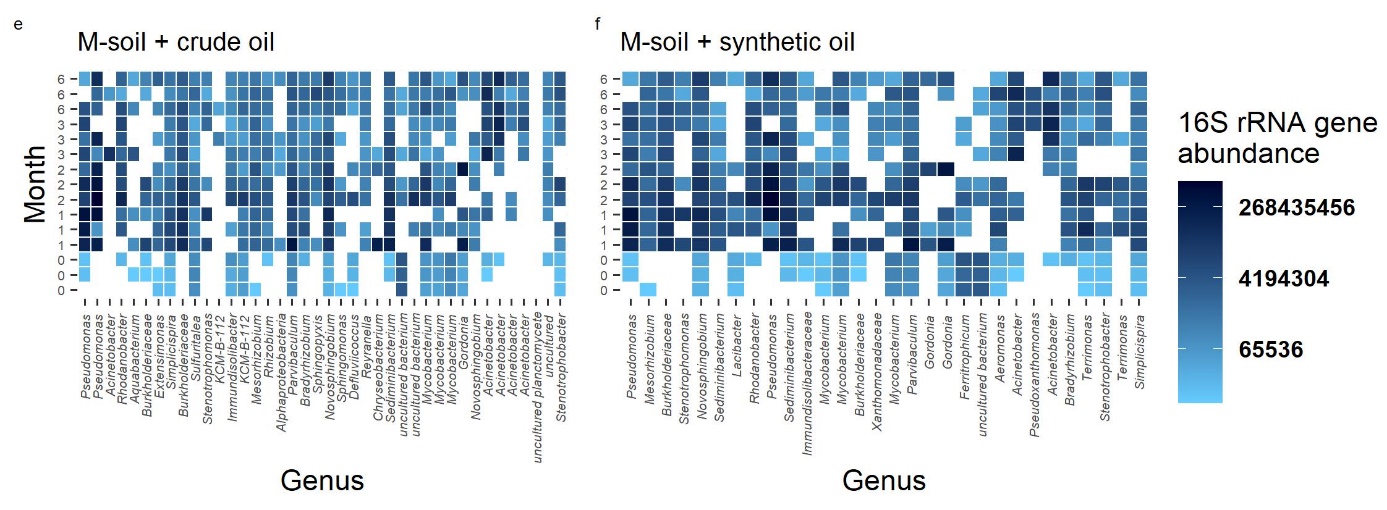

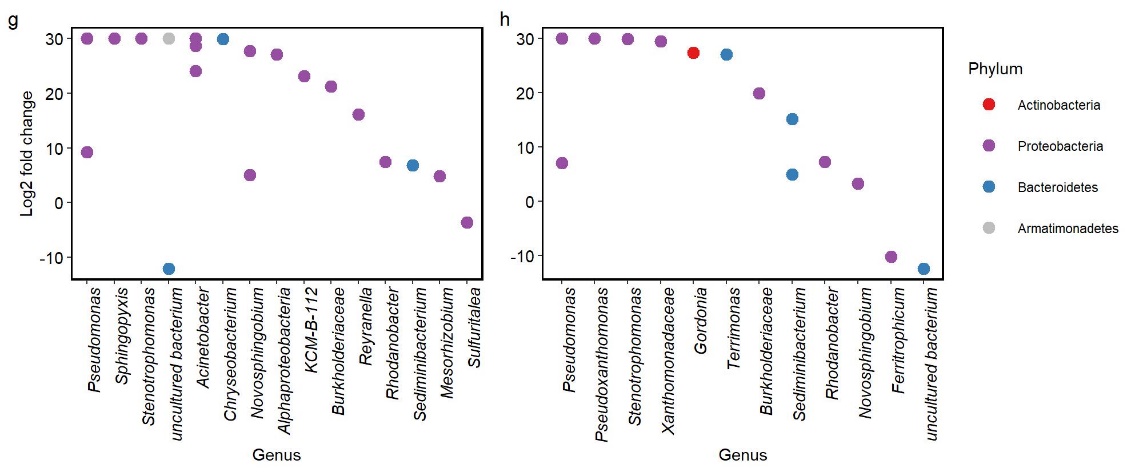


**Supplementary Figure 2.** Heatmaps and corresponding Log_2_ fold change analyses of two oil-contaminated soils incubated in mineral media with crude oil (spiked with EPA-PAHs) and with a synthetic oil. L-soil is a less contaminated soil and M-soil is a more contaminated soil. Heatmaps (a, b, e, and f), based on Jensen–Shannon divergence (JSD) distance method, represent the abundance of each OTU over time during 0, 1, 2, 3 and 6 months of incubation. Log_2_ fold change (c, d, g, and h) is a differential analysis representing each OTU whose abundance significantly (p<0.01) changed over time from time zero versus the following sampling times after the start of the experiment. Taxa with Log_2_ fold change > 0 have increased in abundance while Log_2_ fold change < 0 indicated the abundance of the genera have decreased. *A4b* is an unassigned genus belonging to the *Chloroflexi*. KCM-B-112 is an unassigned genus belonging to the family *Acidithiobacillaceae*.

**
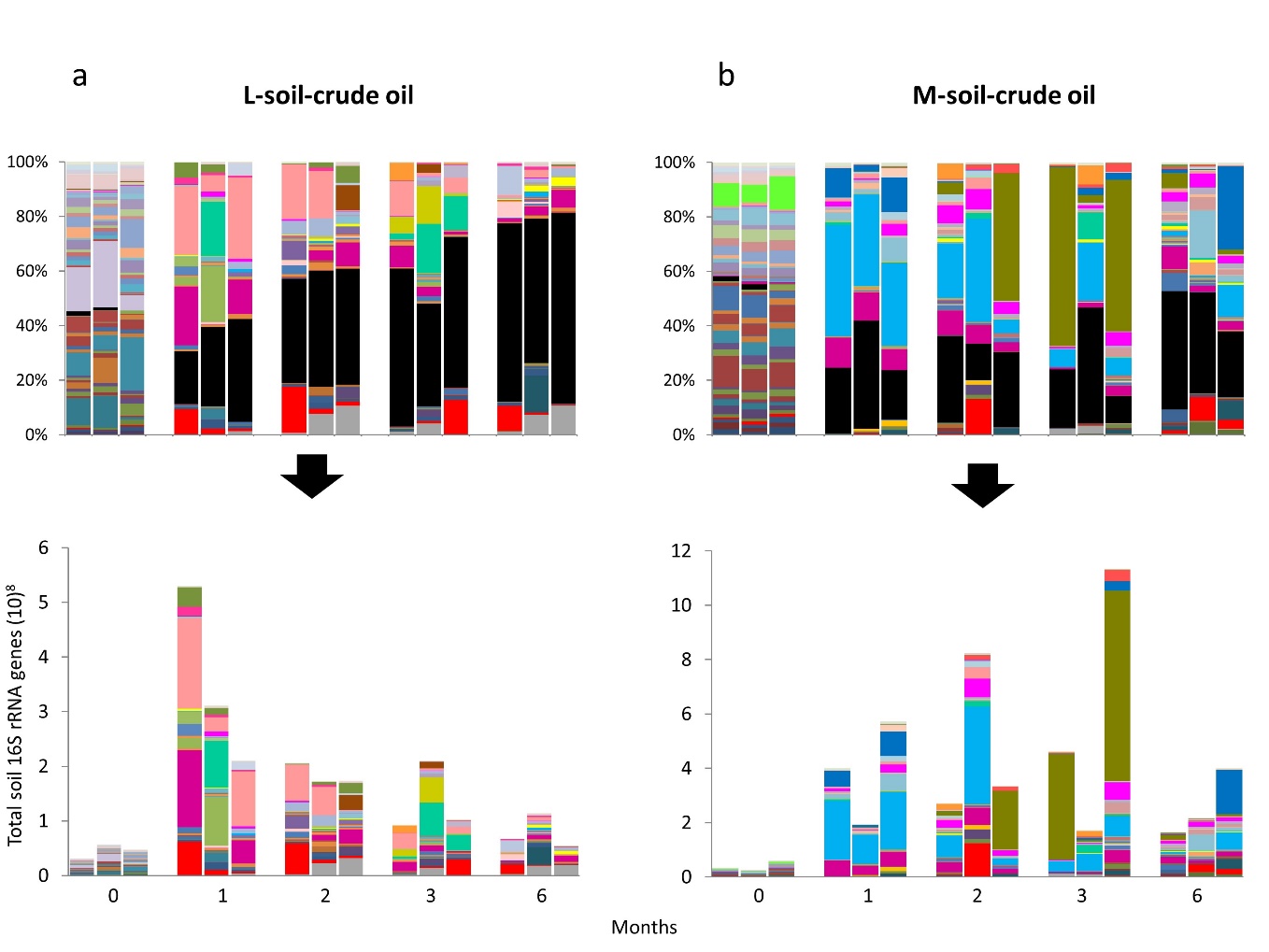

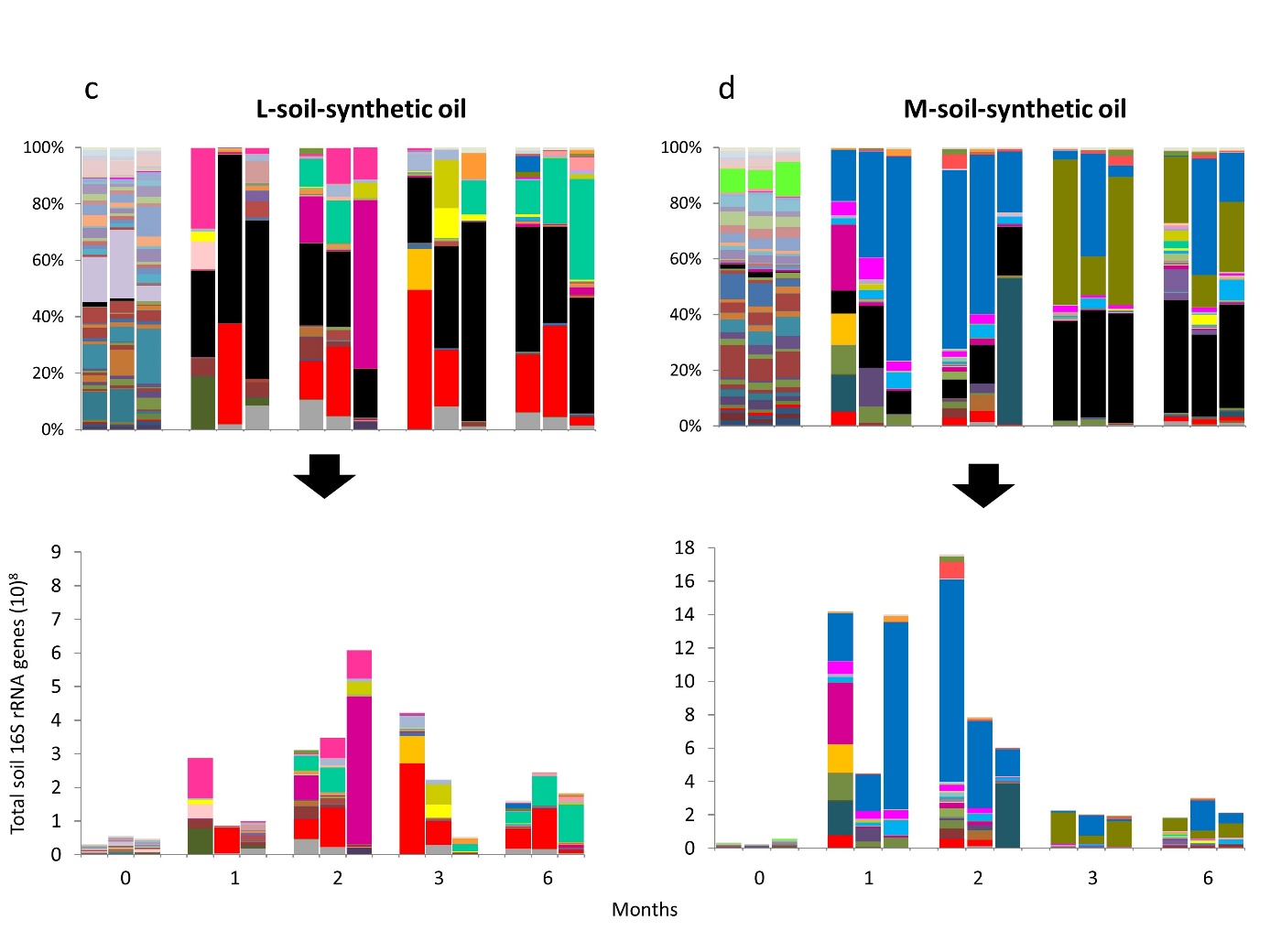
**

**Supplementary Figure 3**. Calculation of Total 16S rRNA gene of incubation enrichments of two soils with different degree of contamination. A more contaminated soil (M-soil) and a less-contaminated soil (L-soil) were incubated with crude oil (a and b) and synthetic oil (c and d). Genomic DNA of *Geobacillus kaustophilus* was added to the samples as internal standard in order to convert relative abundances of the OTUs to quantitative abundances across samples using 16S rRNA gene sequencing. Each color represent the relative abundance of the OTUs at genus level. The abundance of *G. kaustophilus* is in black. Bars are biological triplicates.

**
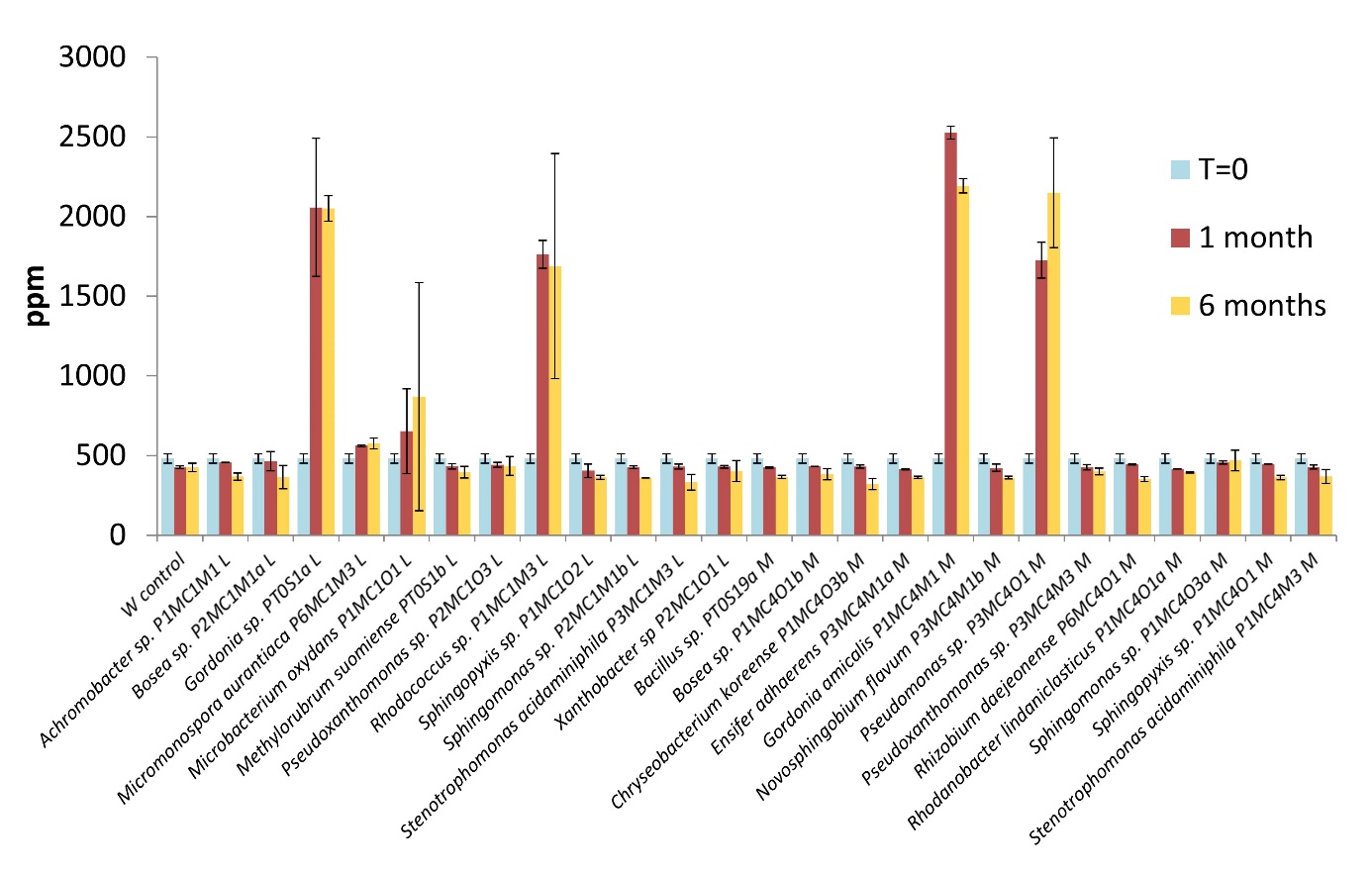
**

**Supplementary Figure 4**. CO_2_ concentration in head space during the degradation of a synthetic oil by single isolates. Cultures were made using 10 mL of medium in 30 mL serum bottles closed with Teflon caps. Incubations were at 30°C for six months. CO_2_ production against a weathering control (W control) was measured by gas chromatography coupled with barrier ionization detector (GC-BID). The species ending with L were isolated from a less-contaminated soil and the species with M were isolated from a more-contaminated soil.

**Supplementary Table 1.** Genera isolated from less-contaminated soil (L-soil). Numbers are the average of triplicates of relative abundance of each genus in the community profile. Grey highlights the genus that has been isolated on agar plates at the sampling times indicated.

|  | **Synthetic oil**  (months) | | | | | **Crude oil**  (months) | | | | |
| --- | --- | --- | --- | --- | --- | --- | --- | --- | --- | --- |
| Genera | 0 | 1 | 2 | 3 | 6 | 0 | 1 | 2 | 3 | 6 |
| *Achromobacter* | 0.000 | 0.003 | 0.000 | 0.000 | 0.000 | 0.000 | 0.000 | 0.000 | 0.000 | 0.000 |
| *Bacillus* | 0.553 | 0.000 | 0.511 | 0.000 | 0.000 | 0.553 | 0.000 | 0.000 | 0.000 | 0.000 |
| *Bosea* | 0.000 | 4.776 | 0.194 | 0.000 | 0.000 | 0.000 | 0.386 | 1.272 | 0.000 | 6.023 |
| *Dyella* | 0.000 | 15.457 | 10.705 | 0.492 | 0.468 | 0.000 | 1.964 | 0.718 | 0.531 | 1.962 |
| *Gordonia* | 0.000 | 0.000 | 0.000 | 0.000 | 0.000 | 0.000 | 0.000 | 0.000 | 0.000 | 9.513 |
| *Hydrogenophaga* | 0.000 | 2.479 | 2.735 | 4.971 | 1.309 | 0.000 | 1.594 | 7.209 | 1.151 | 4.718 |
| *Methylorubrum* | 0.000 | 0.000 | 0.000 | 0.011 | 0.000 | 0.000 | 0.000 | 0.000 | 0.003 | 0.000 |
| *Microbacterium** | 0.000 | 0.127 | 0.338 | 0.000 | 0.446 | 0.000 | 2.145 | 2.276 | 2.448 | 3.133 |
| *Micromonospora** | 0.000 | 0.000 | 0.000 | 0.000 | 0.000 | 0.000 | 0.000 | 0.000 | 0.000 | 0.015 |
| *Pseudoxanthomonas* | 0.000 | 0.049 | 0.972 | 0.000 | 0.757 | 0.000 | 3.617 | 4.823 | 0.000 | 0.680 |
| *Rhodococcus* | 0.000 | 11.158 | 0.000 | 0.000 | 0.058 | 0.000 | 0.000 | 0.000 | 0.080 | 0.000 |
| *Sphingomonas* | 0.000 | 1.735 | 0.192 | 7.981 | 0.846 | 0.000 | 0.306 | 1.003 | 0.131 | 5.007 |
| *Sphingopyxis* | 0.000 | 0.000 | 11.446 | 13.492 | 39.086 | 0.000 | 9.275 | 0.000 | 20.751 | 0.000 |
| *Stenotrophomonas* | 0.000 | 0.000 | 0.000 | 10.204 | 0.809 | 0.000 | 0.000 | 0.000 | 5.523 | 0.000 |
| Thermomonas | 0.000 | 0.000 | 0.000 | 0.000 | 0.611 | 0.000 | 2.435 | 0.000 | 0.000 | 0.248 |
| Xanthobacter | 0.000 | 0.010 | 0.000 | 0.007 | 0.000 | 0.000 | 0.032 | 0.061 | 0.003 | 0.017 |

* In the 16S rRNA gene community profile this genus was identified only at family level (*Microbacteriaceae* or *Micromonosporaceae)*.

**Supplementary Table 2.** Genera isolated from more-contaminated soil (M-soil). Numbers are the average of triplicates of relative abundance of each genus in the community profile. Grey highlights the genus that has been isolated on agar plates at the sampling times indicated.

|  | **Synthetic oil**  (months) | | | | | **Crude oil**  (months) | | | | |
| --- | --- | --- | --- | --- | --- | --- | --- | --- | --- | --- |
| Genera | 0 | 1 | 2 | 3 | 6 | 0 | 1 | 2 | 3 | 6 |
| *Bacillus* | 1.194 | 0.000 | 0.000 | 0.000 | 0.059 | 1.194 | 0.000 | 0.000 | 0.000 | 0.000 |
| *Bosea* | 0.000 | 0.000 | 0.073 | 0.162 | 0.203 | 0.000 | 0.182 | 0.188 | 0.272 | 0.223 |
| *Chryseobacterium* | 0.000 | 4.056 | 0.041 | 0.000 | 0.000 | 0.000 | 1.408 | 0.628 | 0.039 | 0.000 |
| *Gordonia* | 0.000 | 4.830 | 21.160 | 0.000 | 0.990 | 0.000 | 0.535 | 0.941 | 0.539 | 3.834 |
| *Hydrogenophaga* | 0.172 | 0.111 | 0.081 | 0.000 | 0.282 | 0.172 | 0.041 | 0.000 | 0.391 | 0.223 |
| *Novosphingobium* | 0.056 | 4.153 | 2.847 | 2.019 | 4.581 | 0.056 | 48.884 | 26.640 | 17.277 | 6.647 |
| *Pseudomonas* | 0.000 | 49.591 | 53.801 | 23.889 | 29.228 | 0.000 | 11.297 | 0.279 | 2.672 | 14.274 |
| *Pseudoxanthomonas* | 0.000 | 0.209 | 0.741 | 1.214 | 1.426 | 0.000 | 0.165 | 0.137 | 0.081 | 0.771 |
| *Rhizobium* | 0.000 | 0.073 | 0.077 | 0.161 | 0.165 | 0.000 | 0.362 | 0.154 | 0.213 | 0.613 |
| *Rhodanobacter* | 0.000 | 0.252 | 2.550 | 2.638 | 0.894 | 0.000 | 0.138 | 2.241 | 2.151 | 0.810 |
| *Rhodococcus* | 0.000 | 0.147 | 0.000 | 0.000 | 0.000 | 0.000 | 0.000 | 0.000 | 0.000 | 0.000 |
| *Sphingomonas* | 0.000 | 0.043 | 0.036 | 0.276 | 2.210 | 0.000 | 0.041 | 0.717 | 0.684 | 1.531 |
| *Sphingopyxis* | 0.000 | 0.000 | 0.081 | 0.324 | 1.509 | 0.000 | 0.486 | 1.037 | 5.952 | 0.978 |
| *Stenotrophomonas* | 0.000 | 1.024 | 0.362 | 0.166 | 0.200 | 0.000 | 0.000 | 2.714 | 4.135 | 0.000 |
| *Ensifer* | 0.000 | 0.000 | 0.000 | 0.000 | 0.000 | 0.000 | 0.000 | 0.000 | 0.000 | 0.000 |
